# High Retention and Purification of Bromelain Enzyme (*Ananas comosus* L. Merrill) from Pineapple Juice Using Plain and Hollow Polymeric Membranes Techniques

**DOI:** 10.3390/polym14020264

**Published:** 2022-01-10

**Authors:** Felix M. Carbajal Gamarra, José C. C. Santana, Segundo A. V. Llanos, Jorge A. Heredia Pérez, Fábio Richard Flausino, Ada P. B. Quispe, Pedro Córdova Mendoza, Rosangela M. Vanalle, Carmen Carreño-Farfan, Fernando T. Berssaneti, Roberto R. de Souza, Elias B. Tambourgi

**Affiliations:** 1 Energy Engineering, University of Brasilia, FGA-UnB, St. Leste Projeção A—Gama Leste, Brasilia 72444-240, DF, Brazil; 2 Department of Management Engineering, Federal University of ABC, University Mall, São Bernardo do Campo 09606-045, SP, Brazil; jose.curvelo@ufabc.edu.br; 3 Facultad de Ingeniería Química e Industrias Alimentarias, CYMAIDS, Universidad Nacional Pedro Ruiz Gallo, Calle Juan XXIII 391, Lambayeque 14013, Peru; svasquezll@unprg.edu.pe (S.A.V.L.); abarturen@unprg.edu.pe (A.P.B.Q.); 4 Business School, Universidad del Pacífico, Calle Sanchez Cerro 2141, Jesús Maria, Lima 15072, Peru; ja.herediap@up.edu.pe; 5 Industrial Engineering Postgraduate Program, Nine July University, Vergueiro Street, Liberdade, São Paulo 01504-001, SP, Brazil; fabiorflausino@gmail.com (F.R.F.); rvanalle@uni9.pro.br (R.M.V.); 6 Facultad de Ingeniería Ambiental y Sanitaria, Universidad Nacional San Luis Gonzaga de Ica, Ciudad Universitaria, Km 305, Ica 11004, Peru; pedro.cordova@unica.edu.pe; 7 Facultad de Ciencias Biológicas, CYMAIDS, Universidad Nacional Pedro Ruiz Gallo, Calle Juan XXIII 391, Lambayeque 14013, Peru; ccarreno@unprg.edu.pe; 8 Department of Production Engineering, Polytechnic School of State University of São Paulo, Av. Prof. Luciano Gualberto, 1380—Butantã, São Paulo 05508-010, SP, Brazil; fernando.berssaneti@usp.br; 9 Department of Chemical Engineering, Federal University of Sergipe, DEQ/UFS, University Campus “José Aloísio de Campos”, Av. Marechal Rondon, S/N, Rosa Elze, São Cristóvão 49100-000, SP, Brazil; rrsouza@ufs.br; 10 School of Chemical Engineering, State University of Campinas, DESQ/FEQ/UNICAMP, University Campus “ZeferinoVaz”, Av. Albert Einstein, 500, Campinas 6066, São Paulo 13083-840, SP, Brazil; ebt@feq.unicamp.br

**Keywords:** pineapple, bromelain, separation process, hollow fiber membrane, plain membrane, enzymatic activity

## Abstract

The demand for bromelian and pineapple fruit has been increasing substantially in the world because of their benefits for the human health and use in diverse areas. In this context, this work aimed to study the capacity of higher retention (concentration); bromelain activity underwent ultrafiltration from pineapple juice (*Ananas comusus* L. Merrill). All assays were carried out at pH 7.0 and 7.5, and at 0.05 and 0.40 bar of transmembrane pressures. Results have shown that at the best operating conditions, between 85 and 87% of bromelain activity was recovered using the plain membrane separation process at 0.05 bar. The ultrafiltration has shown the capacity to retain 100% of proteolytic activity of the bromelain extracted. The samples have kept the same physics properties after ultrafiltration, and the result was verified via electrophoresis. The bromelain enzyme obtained was characterized, and pH 7 and between 30 and 40 °C were the best conditions. Therefore, this work shows that the use of both polymeric membranes has shown high efficiency, and can be used in the purification of bromelain enzymes.

## 1. Introduction

The production of pineapple fruit (*Ananas comosus*) has increased sustainably during the last two decades. However, in the last decade, the demand for this fruit has expanded, particularly for Asian households. Pineapple can be consumed in several ways, such as in nature form, fresh-cut preserved fruit or mixed with flavor foods such as beef, ham or chicken meat. Additionally, in the last ten years, an important change in consumer preferences was observed, being that fresh *Ananas* fruit was more in demand than canned [1,2]. Pineapple has been important in human food for over five centuries, but it was only after the discovery of pineapple bromelain that it increased its economic interest and boosted research into the purification and use of this enzyme [2,3,4,5].

In essence, several benefits are founding in the bromelain enzyme from pineapple in various areas. In the medical contribution this enzyme is associate with cystein protease activity. This enzyme shows fibrinolytic, anti-inflammatory, thrombolytic and edematous activities [2,3,4,5,6], which, as well, have been applied in the treatment of enkephalin [7] and leukemia, in anticancer activity [8], in the treatment of allergic airway disease [9,10,11] and in cosmetic compositions [12,13]. Bromelain has been used for dental care, in digestive aid and in food industries, such as breweries, meat processing, textiles and other [14,15,16,17,18,19]. Therefore, because the important benefits found for the health of humans and properties mentioned, commercial bromelain reaches a high price, being sold at USD 1000 per kg [4]. Additionally, as the proteinase complex derived from pineapple, bromelains are more stable when they are found in concentrated solutions; mainly, pH (6.5–7.5) and 37 °C are considered to be the best condition [5,20,21].

In this line, aqueous two-phase systems (ATPS) have been used since the 1950s in the separation of biomolecules from importance for biotechnology, food and medicine, as it is effective, non-toxic, easy to handle and economically viable [18,22,23,24,25]; however, this is best suited as a pre-purification method. In this context, bromelain needs to be purified, being in a continuous aqueous two-phase system (ATPS), which is further indicated in the literature. Under this system, we found a 3-fold purification factor using a polymer with reverse micelles in the organic phase [22]. When an ATPS with a reverse micelle polymer was used to extract bromelains from the pineapple peel, a 2.7-fold purification factor was been found, demonstrating a similarity between the efficiency of the continuous and batch ATPS processes [23]. In this line, the pineapple bromelain was purified using a ATPS with metal chelate ionic liquid-based in a flotation system (IL-based ATPF) and a two-step precipitation process, found a purification factor of 6.56-folds [26]. However, a 13-fold purification factor was found for expanded bed chromatography, which was used to purify pineapple bromelain by adsorption on an ion exchange resin [27].

Transport phenomena in membrane processes have been studied theoretically with lab-scale membrane modules continuously fed with fruit juice, beer, wine and other beverages. In a wide range of shear rates, these may be considered as non-Newtonian fluids behaving rheologically, according to a power-law equation. The dependence of apparent viscosity and, therefore, of the diffusion coefficient on concentration has been taken into account [28]. The concentration polarization is a phenomenon, typical of membrane operation, that must be maintained within acceptable limits. Hydraulic permeability of the membrane, generalized Reynolds number and fluid rheological properties were shown to play a crucial role in system performance. Both the diffusion coefficient and the free stream velocity are critical in evaluating membrane performance. For the decrease in permeate flux the opposite trend between the diffusion coefficient and axial velocity is observed at a fixed Reynolds number, due to the fouling effect [28]. It increased due to the fouling effect and the reduction in permeate flux. According to Barros et al. [5], the permeation rates are very low when fruit juices are used, and the main reason for this behavior is the passage into membrane pores by the small molecules obtained from the breakage of great molecules, causing clogging of the hollow fiber polysulfone membrane channels. The concentration by polarization reduces the hydraulic permeability of the membrane and its occurrences decrease the membrane performance. The decrease in permeate flux reduces the diffusion coefficient [1,16].

Advantages offered by membrane process clarification are: a reduction in clarification times; the simplification of the clarification process; an increase in the amount of clarified juice; the possibility of operating at room temperature and preserving juice freshness, aroma and nutritional value; an improvement in the quality of the final product through removal of extraneous substances and an improvement in the production process [28,29].

This makes it possible to maintain and even improve the quality of food, medicinal and biotechnological products; because membrane technology retained their color, carbohydrates, sugars, proteins and most of their aroma, which allows for obtaining a concentrated juice of high quality and high nutritional value [28,29].

The fact, the membranes separation process is one of the most applied emergent technologies in the last two decades to obtain biomolecules. This process used a filtering polymer membrane, which behaves as a physical barrier that restricts partial transporting into phase of various chemical species by its size [28,29,30,31,32]. The separation occurs when, the crude solution containing the target biomolecule flux through the pores of a filtering membrane, which controls the species transporting rate, getting a poor and a rich phase in the target biomolecule [31,32]. The most common polymers membranes are the flat and hollow fibers of microfiltration (MF) and ultrafiltration (UF) and reverse osmosis (RO) [30,32,33,34,35,36,37,38,39,40]. Models to describe the fluid dynamic behavior of biomolecule microfiltration processes have been developed for decades, such as the models presented by cite the behavior with non-Newtonian fluids; the loss of function/activity of the target molecules due to the action of high pressures; breakage, breaking of bonds or formation of isomers due to forced passage between the pores of the filtering membrane. So, to study the best way to obtain these biomolecules without losing their functionalities is of great importance for biotechnology, medicine, food, chemical, pharmaceutical industries.

Currently, there is no report on the purification of bromelains showing which are the best filtering membranes in terms of their shape (flat or roll) and pressure variation in order to achieve a high degree of purity. In this context, this work aimed the capacity of higher retention (concentration) the bromelain activity by membrane separation process from pineapple juice (*Ananas comusus* L. Merrill) to compare the efficiencies of microfiltration process used with of flat and hollow fiber membranes. Membrane filtration is a clean technology that does not interact with the products, with reduced consumption of water and chemicals; with extended life, resulting in less maintenance and downtime, which makes its cost low. The selected polymeric membranes are efficient, and have an advantage over the others, due to the ease of their use on an industrial scale [30,31]. In this direction, this work has evaluated the recovery of bromelain from pineapple via the membrane separation process, and after purification, the enzyme was bio-characterized.

## 2. Materials and Methods

### 2.1. Material

Comassie brilliant blue G (98% purity), HCl PA (37% purity), NaOH PA (99.5% purity), Na_2_HPO_4_ PA (98% purity), KH_2_PO_4_ PA (99.0% purity), Tris(hydroxymethyl) aminomethane (99.5–100% purity) were provided by MERCK (Darmstadt, Germany). The pineapple fruits (*A. comosus* L. Merril) were purchased at the municipal supply center in Sorocaba, SP, Brazil.

### 2.2. Methods

#### 2.2.1. Pineapple Juice Extractions

All pre-treatments including (cleaning, cutting and preparing juices) of pineapples were carried out for DESQ Lab of State University of Campinas. The raw juices were stored in refrigerators at 5–10 °C and used for up to one week. The pineapple juice was extracted, prepared at room temperature and pressure, using a pulp mass of 650 g that initially was passed to simple filtration through cotton to remove the dispersed solids. Phosphate buffer at pH 7.0 and 7.5 were used. The solution volume was adjusted to 1.0 L. All other volumes were prepared at this standard concentration (650 g/L) [41,42]. The extract contains same characteristics found in the literature and was used it as references [2,5,27].

#### 2.2.2. Enzyme Assays

Total protein and enzymatic activity were measured in crud, permeate and concentrate samples using Bradford, Murachi and Baldini methods, respectively. The total protein concentration was determined by the Bradford method, in which the absorbance of the samples at 595 nm was compared with a BSA calibration curve [43,44,45,46,47,48,49]. Enzymatic activity method was described by [2,5,22,27], which is based on the enzymatic hydrolysis of 1.2% (*w*/*v*) casein at pH 5 and 35 ± 2 °C for 20 min, followed by the pre-precipitation of the non-hydrolyzed substrate with 5% trichloroacetic acid and analyzed at 280 nm. All analyses were performed in a spectrophotometer UV/Vis of 432 C model, manufactured by FEMTO (São Paulo, Brazil) [49,50]. The efficiency of enzyme recovery was measured using the activity yield (Y%) (Equation (1)) and fold between sides of membrane (Equation (2)). All analyses were released in triplicate for all assays.

(1) $$Y(\%)=\left(\frac{specific\;activity\;of\;global\;permeate}{specific\;activity\;of\;crude}\right)*100$$

(2) $$Fold=\frac{SA_{global}}{SA_{crude}}$$

while SA is specific activity in global permeate or concentrate flux and crude samples.

#### 2.2.3. Polymeric Membrane Separation Processes

In this work, the purification efficiency of plane and hollow fiber microfiltration membrane modules had to be compared. Therefore, two microfiltration membranes of 0.1 µm of pores size were used. The laboratory scale module with a flat polymer membrane consisted of a polyvinyl fluorite plain membrane (0.0225 m^2^ surface area) attached between two acrylic plates with fluid circulation channels and flux and pressure meters at its flux inlets and outlets. All the values shown below are the averages resulting from three or more measurements. Additionally, their respective standard deviations were assumed as a measure of error [49,50,51,52].

The laboratory scale module with a hollow fiber membrane consisted of a polysulphone hollow fiber membrane (0.03 m^2^ surface area) inside an acrylic tube with channels for fluid circulation and pressure meters at its flux inlets and outlets. Scale lab plant operated at full circulation regime (permeate and retentate) for a total volume of 2 L of pineapple juice. Only the filtered pineapple juice (concentrate) was collected in a separate tank [30,31,53]. The filter membrane modules were cleaned in the same way, following the steps presented below. After each assay, passed a stream of distilled water through the membrane modules for 30 min at home temperature (27 ± 2 °C). Thereafter, a flux 0.01 M NaOH solution for 60 min at 40 °C was passed between the membrane, to remove the dirt trapped in their pores and finally washed with distilled water for 20 min at home temperature [32,33,53]. The initial and final permeabilities were used as a parameter to determine the complete cleaning of the filter membranes. The permeability is used to quality control measuring whether the membrane achieves initial conditions [32,33,53]. The essays were made in the DESQ Lab of State University of Campinas. 

All assays were carried between pH 7.0 and 7.5 due to the optimum condition of bromelain from pineapple being in this pH range. For enzyme purification by the plain membrane separation process the filtration were carried out at 0.05 and 0.15 bar of filtration pressures, due to the fragility of this membrane on effect of high pressures. However, for the hollow fiber membrane separation process the filtration was carried out at filtration pressures of 0.10 and 0.40; for other low pressures, the filtration process is not possible. After the experiment’s conclusion, each membrane was placed into an isopropyl alcohol solution, but this solution was not used washing process [30,34,35]. All membranes were put through a pressure meter (manometer), Bourbon C model manufactured by LUBEFER (São Paulo, Brazil).

During all filtrations, 50 mL of permeates were collected and their times to collect were measured. Additionally, as a parameter for finishing the process, a volumetric concentration factor equals to 5 was used in the concentrate flux. This way, as the initial volume was 1 L, so the final volume in the concentrate tank was 200 mL [34,35].

**Figure 1:**
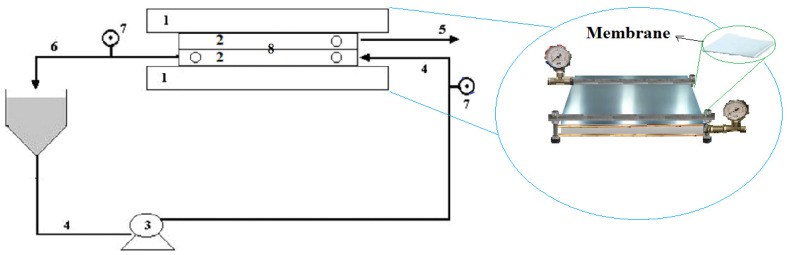
Scheme and photo of the microfiltration unit used in experiments (1—Iron plates; 2—Acryllic plates; 3—Pump; 4—Feed flux; 5—Filtration flux; 6—Concentrate flux; 7—Pressure meter; 8—Membrane).

Polymeric plain membrane system: This is made of polyvinyl fluorite membrane (6501 model; manufactured by TECH-SEP; Aquatech group, Canonsburg, PA, USA). The polymeric membrane used had an area of 0.0225 m^2^, was asymmetrically porous and had a pore size equal to 0.1 μm. The assays were made in a polymeric membrane module composed of two flat props, and was placed between two spacers. In this system, the feed flux circulation occurs in form tangential to the membrane surface, with an interval of time and the respective recirculation to improve the concentration. Figure 1 shows the design of system experimental for the microfiltration unit [30,53]. The concentrate flux occurred through the underside of the flat membrane; nonetheless, the permeate flux occurred over the flat membrane [34,35].

**Figure 2:**
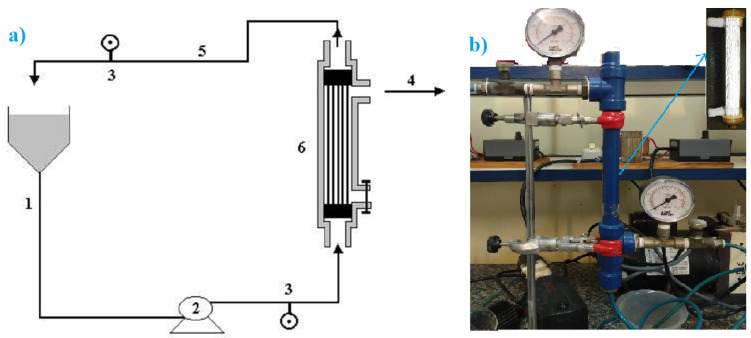
Experimental MMHF-01 Microfiltration Unit (1—Feed flux; 2—Pump; 3—Pressure meter; 4—Filtration flux; 5—Concentrate flux; 6—Hollow fiber membrane). (**a**) Scheme and (**b**) photo of the filter membrane system.

Polymeric hollow fiber membrane system: This is made of a module of polymeric membrane of hollow fiber (MMHF-01-model), shown in Figure 2. The material of membrane used is a polysulfone composite (H1MP0143 model; manufactured by AMICON; Sigma Aldrich group, San Luiz, MI, USA), and with 55 hollow fibers and characteristics of 0.03 m^2^ area, is asymmetrically porous and 0.1 μm of pore size. In this experiment, the pressure and pH were monitored, and the flux of pineapple juice feed was kept in tangential circulation to the membrane surface, until the process was finished [30,31]. This experimental system is shown in Figure 2.

Ultrafiltration: the mixture underwent a second purification step to concentrate the protein content and enable the identification of protein bands on the SDS-PAGE. The best permeated with high bromelain activity was put into a 10 kDa millipore Amicon^®^ propurification system (Merck, Darmstadt, Germany), and the ultrafiltration (UF) process was carried out at 4 °C and 7000 rpm for 20 min. Salts, glycosides and other substances of small molecular weight were eliminated. Total volume of retentate was reduced to 10 times after UF process [27,53].

#### 2.2.4. Resistances Calculations 

Methodologies for calculation of resistances and flux were used according to [29,30,31,45]. Equation (3) determined the permeate flux:

(3) $$J_{v}=\frac{A_{p}}{\Delta t.A_{m}}$$

where permeate flux, L/m^2^·h = filtered volume (L)/membrane area (m^2^). (time variation ∆ (min)).

Membrane resistance ($R_{m}$) used in full experiments was determined with distilled water, according to Equation (4) [30,31]:

(4) $$J_{W}=\frac{\Delta P}{R_{m}}$$

where $J_{W}$ = filtration flux for clean membrane, L/m^2^·h; ΔP = filtration pressure, bar. 

Fouling resistance ($R_{f}$) is equal to resistance sum due to absorption ($R_{a}$) and resistance due to the pores blocking ($R_{b}$). In the completed experiments, $R_{f}$ was considered to be membrane resistant after the filtration experiment, using distilled water, as was shown in Equation (5) [30,31]:

(5) $$J_{W}=\frac{\Delta P}{R_{f}}$$

#### 2.2.5. Enzyme Characterization

The enzymatic activity was measured from permeate and the concentrate using the method developed by Murachi and Baldini. The enzymatic activity was measured in permeate and in the concentrate, using the method described by [22,23,27,44,45,46,47,48,49]. One unit of enzymatic activity was defined as the variation of one absorbency unit at 280 nm during 10 min at 35 °C [2,5].

✓ pH effects: the optimum pH of the enzyme was determined measuring its activity, per 10 min, in the pH varied of 3.5–9.5 at 35 ± 2 °C, using a 5 mg/mL BSA solution in the following media: 0.1 M acetate (pH 3.5–5.0), 0.1 M phosphate (pH 5.5–7.5) and 0.1 M ammonium (pH 8.0–9.5) buffers [2,5].
✓ Temperature effects: the optimum temperature of bromelain was obtained measuring its activity, per 10 min, with temperature range from 10 to 50 ± 2 °C, using some BSA solution in 0.1 M acetate buffer at pH 7.5 [2,5].
✓ Determination of the kinetic constants: initial rates of protein hydrolysis were determined at various substrate concentrations (0.01–5 mg/mL) [2,5].
✓ The kinetic constants *K_m_* and *V_max_* were estimated using the Michaelis–Menten equation (Equation (6)), which was linearized (Equation (7)) by the Lineweaver–Burk method [2,5].

(6) $$V_{0}=\frac{V_{max}\left[S\right]}{K_{m}\left[S\right]}$$

(7) $$\frac{1}{V_{0}}=\frac{1}{V_{max}}+\frac{K_{m}}{V_{max}}\frac{1}{\left[S\right]}$$

✓ Molar weight determination: SDS-PAGE was performed on mini-PROTEAN II cell (Bio-Rad, Hercules, CA, USA) with 12% acrylamide gel, using protein standard as a molecular weight marker.

Proteins of UF extract were separated on 0.8 mm thick homogeneous 12% (*w*/*v*) acrylamide resolving gels and 4.8% (*w*/*v*) acrylamide stacking gels with the buffer systems described by [26] using the Bio-Rad Protean II apparatus. Equal volume of sample buffer that contained 25 mM Tris/HCl, pH 6.8, 20% (*v*/*v*) glycerol, 8% (*w*/*v*) SDS and 0.04% (*w*/*v*) brome-phenol blue, was added to the protein sample and mixed with 2.5% (*v*/*v*) 2-mercapttoethanol. This mixture was boiled for 10 min prior to loading on the gels. The proteins were separated at constant amperage of 20 mA using the running buffer contained 25 mM Tris, 192 mM glycine, and 0.1% (*w*/*v*) SDS, pH 8.3. Separated proteins were visualized after fixation with coomassie brilliant blue G-250 (staining solution: 10% (*v*/*v*) phosphoric acid and 0.02 (*w*/*v*) coomassie) [26]. All the values presented in the following tables are results of data analyzes performed in the Statistica 10 software for Windows^®^ [49,50,53,54,55].

## 3. Results and Discussion

### 3.1. Chemical Characterization

The pulp of pineapple fruit (% dry matter) showed similar characteristics to those found by [23,45]. The protein contain was about 4.8%; however, the pH was very near to 4. The same value was found in the literature [20,23,45], and this low pH indicates the presence of the acid chemical compound, such as malic and citric [32]. Additionally, [45] shows that the characteristics found in the pulp of pineapple is similar to the skin, and the amount of protein is the same with all parts of the fruit. Many of these proteins were insoluble in pineapple juice, which sharply reduced its concentration.

Table 1 below shows the results of the analysis of eight samples of pineapple and their respective juices. Each sample was analyzed in triplicate, and the results are the means and standard deviations of 24 analyzes.

### 3.2. Separation by Hollow Fiber and Micro Filtration Polymeric Membranes

After completion of the process, the final volume of concentrate was 200 mL for all assays. Therefore, this result shows a standardization of volumetric concentration factor equal to 5.0. Thus, the final content of protein in the concentrate may exceed its initial content, but the relationship between the sum of the masses is maintained. In consequence, the mass balance can be easily obtained using Equation (8).

(8) $$C_{Initial}=\frac{200*C_{Concentrate}+800*C_{Permeate}}{1000}$$

**Figure 3:**
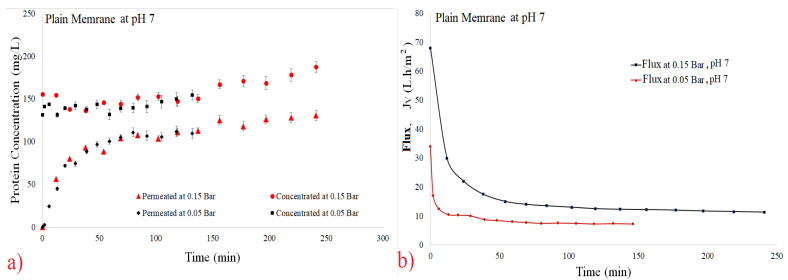
Effect of pressure on filtration flux of the 0.1 µm plain membrane at pH 7. (**a**) Total protein curves and (**b**) flux curves.

**Figure 4:**
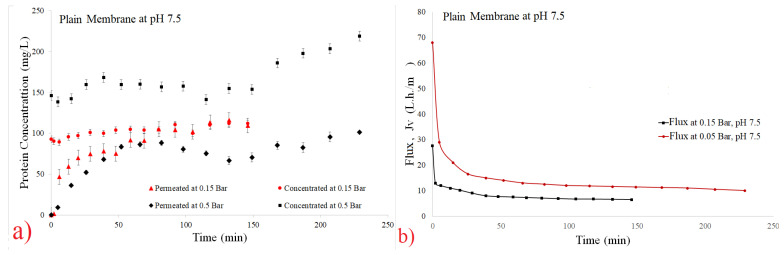
Effect of pressure on filtration flux of the 0.1 µm plain membrane at pH 7.5. (**a**) Total protein curves and (**b**) flux curves.

**Figure 5:**
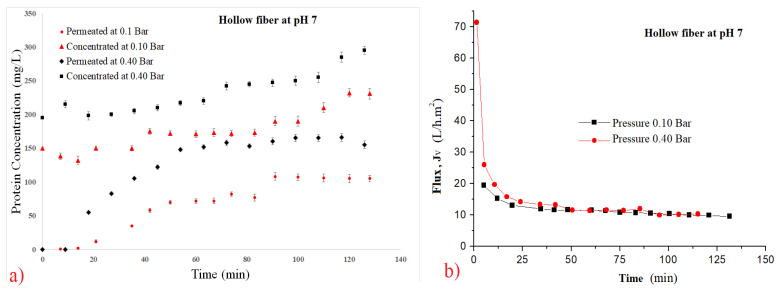
Effect of pressure on filtration flux of the 0.1 µm hollow fiber membrane at pH 7. (**a**) Total protein curves and (**b**) flux curves.

**Figure 6:**
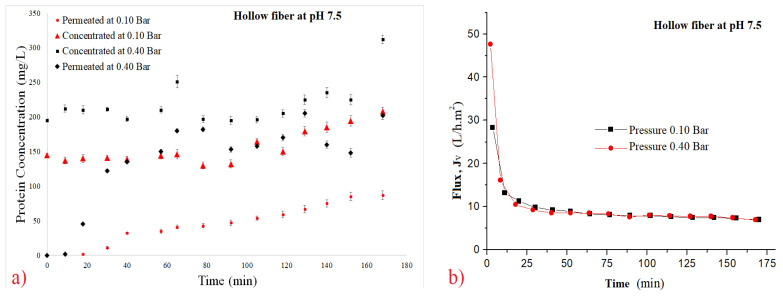
Effect of pressure on filtration flux of the 0.1 µm hollow fiber membrane at pH 7.5. (**a**) Total protein curves and (**b**) flux curves.

Figure 3, Figure 4, Figure 5 and Figure 6 show the filtration flux curve for microfiltration of the pineapple juices for plain and hollow polymeric membrane separation processes at 25 °C. All values presented in these figures are the means of their respective triplicates. It is noteworthy that each experiment was carried out with a different set of pineapple pulp, which provided the difference between the initial concentrations between the tests. In these figures can be observed that a stand state is obtained after 30 min of filtration process [30,31]. Additionally, it is noted that for both filtration pressures, the decrease in flux was high due to the high initial concentration of material in suspension in the juices (pectins, polysaccharydes, tannins, proteins, vitamins, microorganisms, etc.), and it increased due to the fouling effect and the reduction in permeate flux [34,35,39,40]. In addition, the disruption of protein structures is due to breakage of peptide bonds and/or formation of isomers, leading to loss of target protein activity [39].

The permeate rates are very low when fruit juices are used, and the principal reason for this behavior is the passage into the membrane pores by the small molecules obtained from the breakage of great molecules, causing clogging of the membrane channels [30,31]. The problem caused by fouling in the biotechnology industry is due to mechanism of superior protein rejection, which form a layer of slime on the surface of the membrane and can clog its pores [36]. Using ultrasonic waves to mitigate the fouling effect; [37] have been using an antifouling with a branch-like structure of zwitterions grafted onto a membrane via a novel amphiphilic linker [37].

The concentration by polarization reduces the hydraulic permeability of the membrane and its occurrences decrease the polymeric membrane performance. Consequently, the decrease in permeate flux reduces the diffusion coefficient [31,33,53]. Superficial adsorption is another factor which decreases the filtration flux, due to the cake formation [30,31]. In addition, [30] have demonstrated that to insert air in periodic backwash, these resistances can be reduced.

### 3.3. Operation Conditions 

Table 2 can be seen that bout resistances increased the pH and filtration pressure for bout membranes. However, for some pH levels, the difference between the membrane resistance were greater for the plain than the hollow fiber membranes. Additionally, in this table at 0.15 bar, one decrease in clear membrane resistance is noted for the plain membrane; consequently, at 0.05 bar, this resistance was similar. For hollow fiber membranes, these variations were not perceived.

This behavior demonstrated that polysulfone membranes are more selective, and cleaner than polyvinyl fluorite membrane, on the NaOH backwash process. In this line, it was noted that the use of ozonized water in washing processes bettered the membrane performance by 25% more than distilled water, in which the permeate flux increased rapidly for 30 s, then gradually to 70 s [47]. After 70 s, it stayed at about 99.5% of the initial permeate flux and did not increase after that; therefore, the time needed for continuous washing was more than 70 s. It demonstrates that the use of ozonized water is promising in membrane washes.

Table 3 shows the experimental data of bromelain recovery for plain and hollow polymeric membrane separation processes, respectively. The difference amongst the filtration pressures is due to the fact that the hollow fiber membrane needs high pressure for its operation. At low filtration pressures, the hollow fiber had not recovered the enzyme. In this table, it is noted that the plain membrane was more efficient than hollow fiber membranes in bromelain recovery from the pineapple steam. Regarding the best operation condition to bromelain purification by the plain membrane separation process, at 0.05 bar and pH 7 or 7.5, in consequence, it was possible to obtain 85–87% of bromelain activity from pineapple crude extract. Additionally, these behaviors were in relation to lower pressures, resulting in higher purification conditions [29].

The bromelain purification obtained in this work has shown higher capacity than by [22,23,26,27,32,47,56] and similar behavior to [27]. These results were more efficient than the obtained the pineapple bromelain using aqueous two-phase systems in a reverse micelle polymer system [23,57], EOPOEO-based green ATPS [56], PEO-PPO-PEO block copolymers [58] and PEG/Phosphate system [47], which ranged between 10% and 60% of activity recovery; and they were better than the 75% recovery of enzymatic activity obtained by UF at 2.0 bar and 0.30 m/s [29] and were similar to obtained by expanded bad adsorption [29]. which was 12 times greater than the raw pineapple activity.

**Figure 7:**
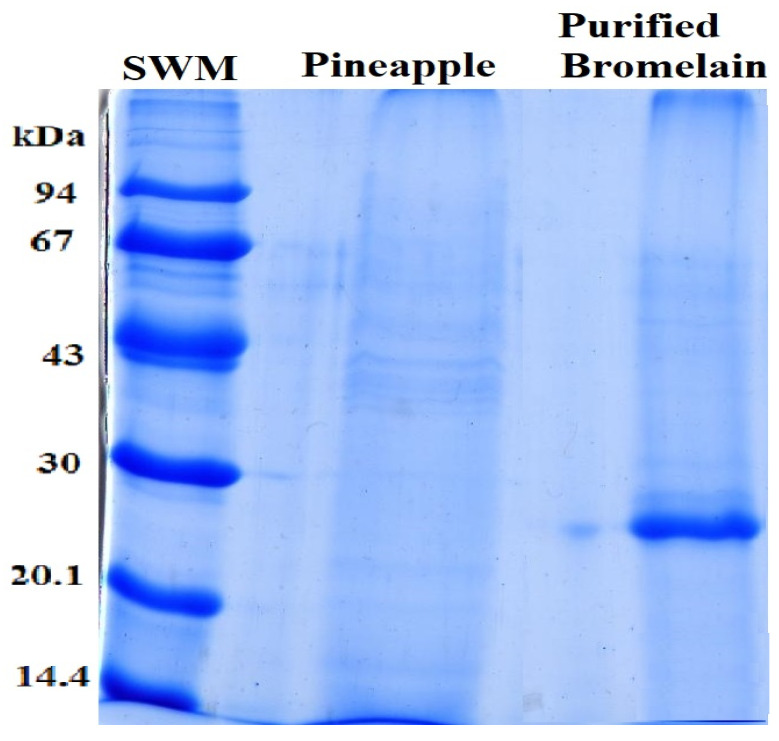
Molecular weight determination by SDS-PAGE electrophoreses.

Impurities such as salts, sugars and other substances with low molecular mass were eliminated from the bromelain concentrated by polymeric plain membrane, and the ultrafiltration was gone; this process retained 100% of proteolytic activity and the enzyme was concentrated 10-fold in relation to the bromelain from the pineapple crude extract. A sample aliquot was collected for the best condition of the plain membrane, and purification was analysed by SDS–PAGE for determination of enzyme molecular weights and degree of purification (see Figure 7) [26,30,45]. This figure shows that the extracted enzyme is pure and their molecular weights of bromelain from *A. comosus* pulp had a molecular weight of 24.5 kDa. This molar mass is about 6% different from the 23 kDa of commercial bromelain and it is close to the Bacillus cereus protease obtained by [58]. A pineapple bromelain of 23 kDa was purifying from Tripura (Indian State), which is a rich source of bromelain along with peroxidase [4]. Additionally, this molecular weight approximated at 28 kDa were found by [20,23,45]. However, it is not similar to the molecular weight of 31 kDa reported in [41] and 32.2 kDa reported by [49]. The bromelain can have various molecular weights; it depends on the original source (e.g., steam or pulp), pineapple variety and harvest [5,49].

From Figure 7, SMW is standard of molecular weight, it is compound of following proteins: phosphorylase b (94 kDa), bovine serum albumin (67 kDa), ovalbumin (43 kDa), carbonic anhydrase (30 kDa), trypsin inhibitor (20.1 kDa) and α-lactoalbumin (14.4 kDa) [26]. The bands with the name pineapple are the sample of pineapple juice before filtration, and the name bromelain is the enzyme purified by the polymeric plain membrane separation process.

**Figure 8:**
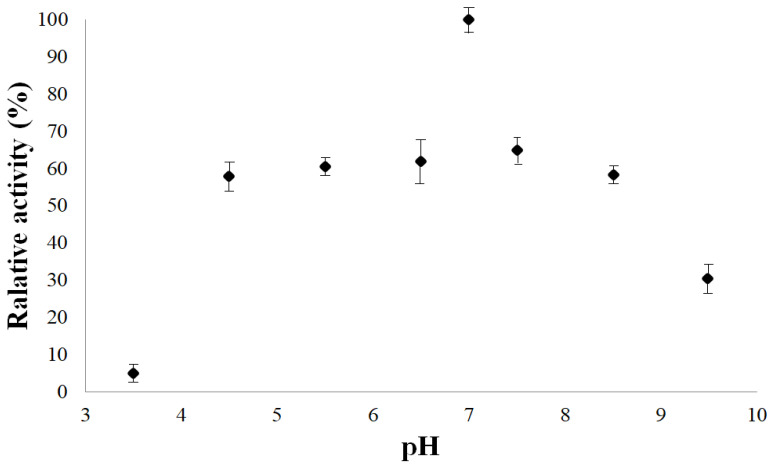
Effect of pH on the bromelain purified from pineapple at 30 °C.

**Figure 9:**
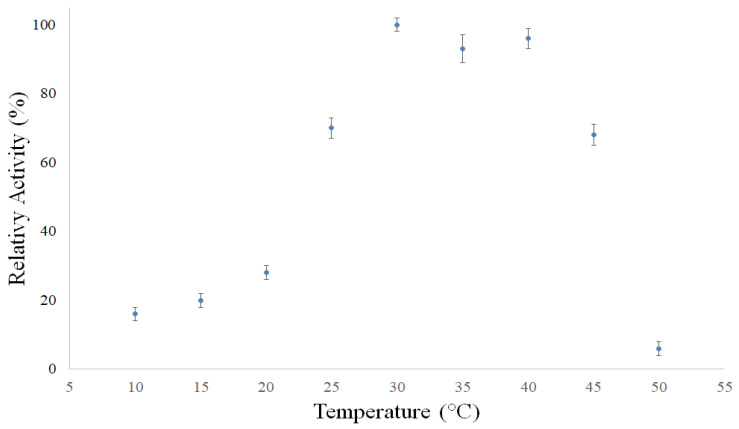
Effect of temperature on the bromelain purified from pineapple at pH 7.

**Figure 10:**
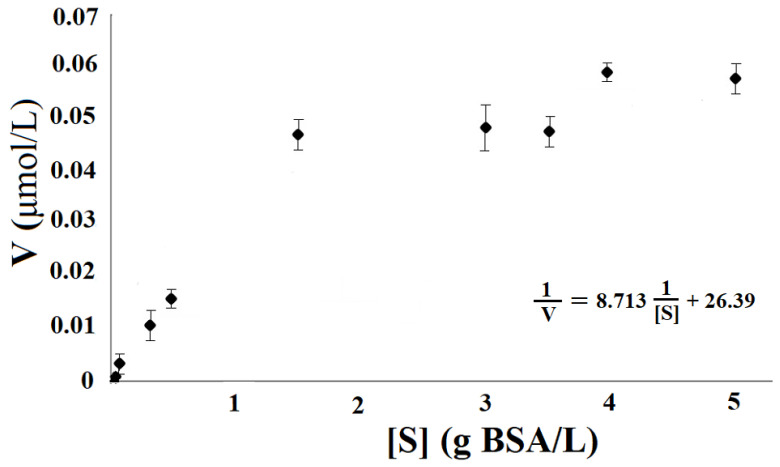
Kinetic of protein hydrolysis by bromelain purified from pineapple at pH 7 and 30 °C.

Figure 8, Figure 9 and Figure 10 showed biochemistry characterization of polymeric bromelain purified from pineapple juice. In these figures, is observed that the optimum pH was about 7, the optimum temperature was between 30 and 40 °C and the *K_m_* and *V_max_* values were of 330.4 mg/L and 2.539 mg BSA/L·min. These optimal pH and temperature were in accordance with those reported by the literature, between 7 and 7.5 [18,48]. However, it is noted that enzyme showed good activity (60%) amongst the pH 4.5 and 8.5; this demonstrates that the pH effect is more minor than temperature effect. This effect on enzyme activities was reliant for the range between pH 4.5 and 8.5, and same for the amylases from maize malt by [43,45,47].

Therefore, the capacity and efficiency was shown for these membranes in order to retain most of the suspended material; it is possible to indicate their applications in the purification of other biomolecules, as nowadays they are used in the clarification of beverages and juices and in the treatment of water and sewage [30,31,57,59].

## 4. Conclusions

The results obtained has shown that, the polymeric plain membrane was more efficient than hollow polymeric membrane in bromelain purification. When transmembrane pressure increased there was reducing of the bromelain activity. At 0.05 bar was the best operation condition to bromelain purification using the polymeric plain membrane, and between 85–87% of bromelain activity was recovered. For ultrafiltration, was possible retained 100% of proteolytic activity and concentrated in 10-fold the bromelain extract. These results were verified via SDS-PAGE electrophoresis, where has shown that, the ultrafiltrated had high purity and the bromelain from *A. comosus* pulp had a molecular weight of 24.5 kDa. The optimum enzyme characterization was *K_m_* 330.4 mg/L and *V_max_* 2.539 mg BSA/L·min for pH 7 at 30–40 °C, and a molecular weight of 24.5 was optimum for the enzyme conditions. The results obtained open the possibility that the small cities with alarge production of pineapple in Latin America can generate a by-product of high aggregate value. Finally, other important contribution in this work have shown that the quality of the purity of the enzymes obtained was superior to that obtained by aqueous two-phase systems, demonstrating a potential alternative to the membrane used for the purification of bromelains.

## Figures and Tables

**Table 1:** Properties of the pineapple pulp and juice at 25 °C.

Analysis	Pineapple Pulp	Pineapple Juice
Moisture, g/100 g	87.2 ± 0.09	92.33 ± 0.14
Protein, mg/L	410 ± 23	115.24 ± 32.99
Density, kg/m^3^	-	1105 ± 98
Viscosity, µ, mPa s	-	1.50 ± 0.21

**Table 2:** Operational conditions after realized experiments.

Assays	pH	Filtration Pressure, ΔP (bar)	Membrane Resistance, *R_m_*, (×10^4^ m^−1^)	Fouling Resistance, *R_f_*, (×10^4^ m^−1^)
Plain membrane	7	0.05	4.83	13.70
	7	0.15	19.90	90.95
	7.5	0.05	4.34	19.80
	7.5	0.15	10.70	154.00
Hollow fiber	7	0.10	8.55	39.26
	7	0.40	12.20	76.12
	7.5	0.10	8.57	45.14
	7.5	0.40	12.15	93.87

**Table 3:** Bromelain purification from *A. comosus* by polymeric plain and hollow fiber membrane, at 25 ± 2 °C.

Process	pH	P (bar)	Sample	Activity, U/mL	Protein, mg/L	SA *, U/mg	%Yields	Fold
Plain membrane			Crud	16.778	131.32	0.128		
	7.0	0.05	Permeate	10.556	98.276	0.109	85.48 ± 1.42	5.74 ± 0.04
			Crud	5004	154.88	0.032		
	7.5	0.05	Permeate	3.031	106.03	0.028	87.21 ± 1.55	6.00 ± 0.05
			Crud	6.134	146.17	0.042		
	7.0	0.15	Permeate	2.668	93.678	0.028	67.87 ± 1.88	2.00 ± 0.03
			Crud	2.864	92.672	0.031		
	7.5	0.15	Permeate	0.627	72.701	0.008	29.7 ± 2.14	0.35 ± 0.01
Hollow fiber membrane			Crud	0.089	149.58	0.595		
	7.0	0.10	Permeate	0.021	72.831	0.288	48.46 ± 123	0.94 ± 0.02
			Crud	0.481	192.40	2.50		
	7.5	0.10	Permeate	0.089	203.88	0.436	17.46 ± 2.34	0.21 ± 0.01
			Crud	0.365	194.98	1.872		
	7.0	0.40	Permeate	0.166	156.46	1.06	56.62 ± 3.45	1.30 ± 0.05
			Crud	0.413	145.52	2.84		
	7.5	0.40	Permeate	0.108	85.615	1.26	44.57 ± 2.75	0.80 ± 0.03

* SA: specific activity.

## Data Availability

The data presented in this study are available on request from the corresponding author.
